# High-grade serous ovarian cancer cell lines exhibit heterogeneous responses to growth factor stimulation

**DOI:** 10.1186/s12935-015-0263-4

**Published:** 2015-12-07

**Authors:** Danielle L. Bourgeois, Karl A. Kabarowski, Veronica L. Porubsky, Pamela K. Kreeger

**Affiliations:** Department of Biomedical Engineering, University of Wisconsin-Madison, 1111 Highland Avenue, Madison, WI 53705 USA

**Keywords:** Ovarian cancer, Growth factors, HB-EGF, NRG1β, IGF1, HGF, HGSOC, Metastasis, Tumor heterogeneity

## Abstract

**Background:**

The factors driving the onset and progression of ovarian cancer are not well understood. Recent reports have identified cell lines that are representative of the genomic pattern of high-grade serous ovarian cancer (HGSOC), in which greater than 90 % of tumors have a mutation in *TP53*. However, many of these representative cell lines have not been widely used so it is unclear if these cell lines capture the variability that is characteristic of the disease.

**Methods:**

We investigated six *TP53*-mutant HGSOC cell lines (Caov3, Caov4, OV90, OVCA432, OVCAR3, and OVCAR4) for migration, *MMP2* expression, proliferation, and VEGF secretion, behaviors that play critical roles in tumor progression. In addition to comparing baseline variation between the cell lines, we determined how these behaviors changed in response to four growth factors implicated in ovarian cancer progression: HB-EGF, NRG1β, IGF1, and HGF.

**Results:**

Baseline levels of each behavior varied across the cell lines and this variation was comparable to that seen in tumors. All four growth factors impacted cell proliferation or VEGF secretion, and HB-EGF, NRG1β, and HGF impacted wound closure or *MMP2* expression in at least two cell lines. Growth factor-induced responses demonstrated substantial heterogeneity, with cell lines sensitive to all four growth factors, a subset of the growth factors, or none of the growth factors, depending on the response of interest. Principal component analysis demonstrated that the data clustered together based on cell line rather than growth factor identity, suggesting that response is dependent on intrinsic qualities of the tumor cell rather than the growth factor.

**Conclusions:**

Significant variation was seen among the cell lines, consistent with the heterogeneity of HGSOC.

**Electronic supplementary material:**

The online version of this article (doi:10.1186/s12935-015-0263-4) contains supplementary material, which is available to authorized users.

## Background

Ovarian cancer is the deadliest gynecological cancer in the developed world, with nearly two-thirds of patients diagnosed with advanced, metastatic disease [1]. Most of these patients initially respond to standard treatment of surgical debulking and chemotherapy, but over 70 % exhibit disease recurrence and eventual chemoresistance. High-grade serous ovarian cancer (HGSOC), a subtype characterized by a mutation in *TP53*, a low rate of other mutations, and extensive DNA copy number changes, is the most aggressive and most common subtype of ovarian cancer, accounting for two-thirds of deaths [2, 3]. Due to the limited animal models that mimic the disease [4, 5], HGSOC has been primarily studied in vitro and through xenograft models with cell lines. However, a recent analysis of ovarian cancer cell lines in the Cancer Cell Line Encyclopedia (CCLE) indicated that the most commonly used cellular models of HGSOC (i.e., SKOV3 and A2780) do not mimic the genomic characteristics of tumors in The Cancer Genome Atlas (TCGA) database and suggested that future research should use more representative lines to develop improved treatment strategies [6]. Extensive patient-to-patient variation is observed within the HGSOC subtype; therefore, it will be important to determine if limiting studies to these representative lines can recapitulate this heterogeneity. To address this question, we performed the first comprehensive study of representative HGSOC cell lines to examine the variation in their baseline and growth factor-induced behaviors with respect to processes that are important in metastasis.

Unlike other solid tumors, ovarian cancer is not restricted to metastasis through the blood and lymph systems; instead, tumor cells can exfoliate from the ovary and attach to organs in the peritoneum, especially the omentum [7]. This peritoneal metastasis is a complex process that relies on many different cellular actions, including migration, extracellular matrix remodeling, proliferation, and angiogenesis. Tumor cell migration is a key step for dissemination of the tumor along the peritoneum [8], and has been modeled in vitro using two-dimensional cell migration assays. In addition to this mechanism of spreading, ovarian cancer cells can detach, transport through the peritoneal fluid and attach to new metastatic sites. The attachment of these cells into their new sites is mediated in part by matrix-metalloproteinase-2 (MMP2) [9]; MMP2 is also responsible for invasion of the tumor deeper into the tissue [10]. Studies have found that MMP2 levels in ascites fluid increase in advanced stage ovarian cancer [11] and overexpression of *MMP2* in peritoneal implants correlates with elevated mortality risk [12]. Following implantation and invasion, continued growth and viability of the tumors is maintained through cell proliferation and angiogenesis. Not surprisingly, advanced stages of ovarian cancer and mortality risk are both associated with high rates of proliferation [13]. As in other solid tumors, angiogenesis in ovarian cancer is mediated by the production of angiogenic factors such as vascular endothelial growth factor (VEGF) that recruit new vessels from the native vasculature [14–16].

The different stages of HGSOC metastasis are influenced by the presence of growth factors and cytokines in the tumor microenvironment, which in HGSOC includes ascites fluid. For example, heparin-binding EGF-like growth factor (HB-EGF), neuregulin-1 beta (NRG1β), insulin-like growth factor 1 (IGF1), and hepatocyte growth factor (HGF) are all expressed in tumors and found at higher levels in ascites fluid of ovarian cancer patients compared to healthy controls [17–21]. Elevated *HB*-*EGF* expression has been associated with shorter progression-free survival [22]; HB-EGF treatment induced invasion and VEGF production by SKOV3 in vitro and promoted peritoneal dissemination of xenografts [23]. Autocrine NRG1β increased cell growth and decreased survival time in several xenograft mouse models of ovarian cancer [21]. Overexpression of *IGF1* was associated with shorter progression-free survival [24] and has been shown to increase proliferation of OVCAR3 in vitro [25]. Elevated serum levels of HGF were exhibited in >90 % of tumors and correlated to shorter overall survival of ovarian cancer patients [26]. In vitro, HGF mediated an epithelial-to-mesenchymal transition and sustained anchorage-independent growth of ovarian cancer cells [27, 28].

Therefore, to determine if HGSOC cell lines that have genomic profiles similar to TCGA tumors (Caov3, Caov4, OV90, OVCA432, OVCAR3, OVCAR4) demonstrate heterogeneity in the various metastatic processes, we examined migration, *MMP2* expression, proliferation, and VEGF secretion in response to HB-EGF, NRG1β, IGF1, and HGF.

## Results

### Tumor cell migration in response to growth factors varied across HGSOC cell lines

**Fig. 1:**
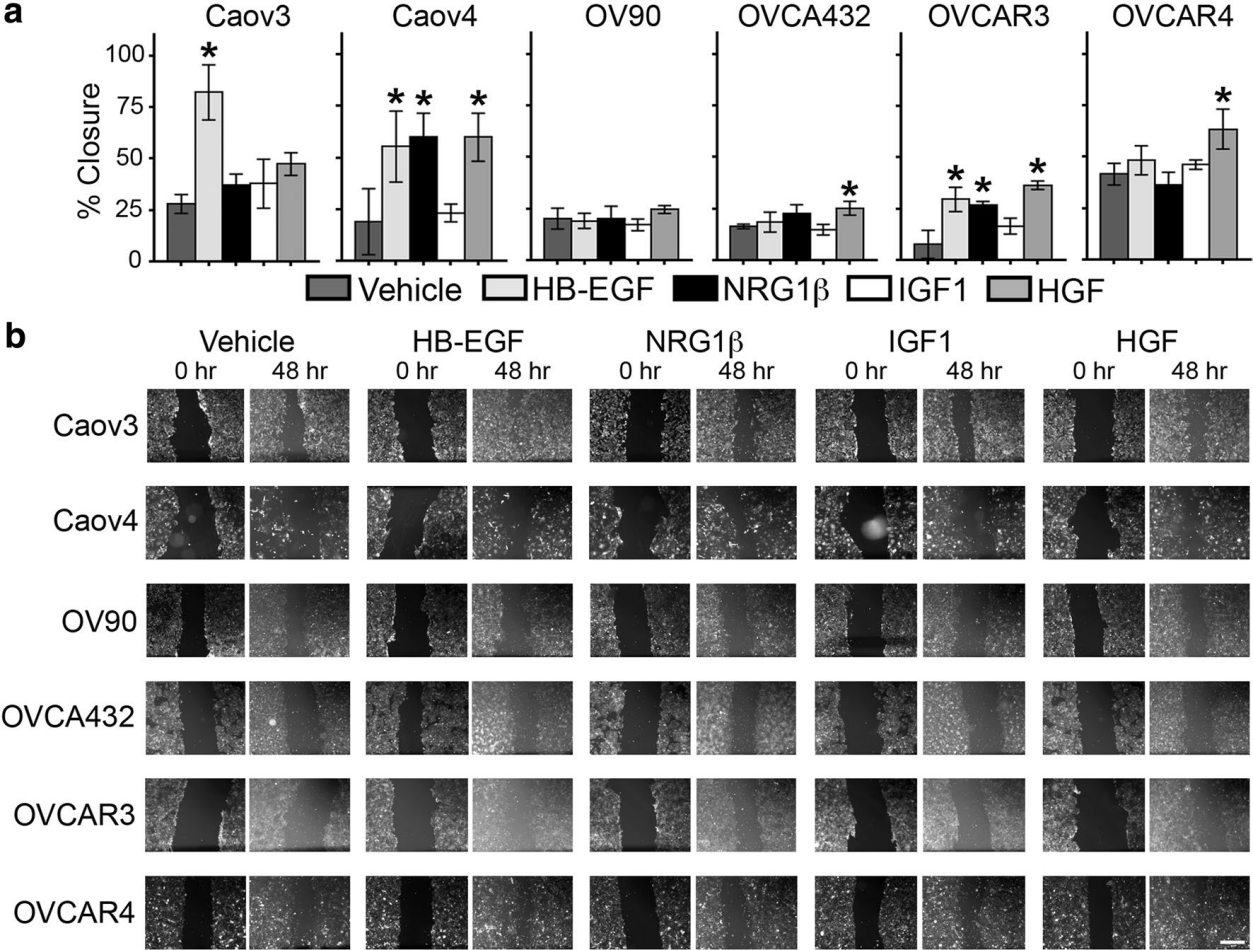
The effects of growth factors on collective migration of HGSOC cell lines. **a** Treatment with 10 ng/mL HB-EGF, NRG1β, IGF1, or HGF for 48 h impacted wound closure in a subset of HGSOC cell lines. Cells were stained with CellTracker Green, seeded to confluency, and wound area was measured at 0 and 48 h post-treatment. Data presented as the average ± SD, n = 3. *p < 0.05 compared to vehicle. **b** Representative images of the wounds at time = 0 and 48 h for each cell line and treatment condition. As expected, CellTracker Green intensity had decreased by 48 h, however, the edge of the wound could still be analyzed. *Scale bar* represents 500 μm

Progression in HGSOC is marked by the dissemination of tumor cells throughout the peritoneum [8], with tumor cells present as both single cells and as aggregates [29]. Therefore, to model the behavior of these different cellular presentations, collective cell migration was examined by wound assays and single cell motility was modeled utilizing transwell assays. In the wound assays, we determined that all six cell lines migrated in the absence of stimulatory factors and that the extent of wound closure varied across the cell lines, ranging from 7.6 % for OVCAR3 to 41.4 % for OVCAR4 (Fig. 1). Following growth factor treatment, we observed that HGSOC cell lines had significantly increased migration after treatment with (1) three of the growth factors (Caov4, OVCAR3), (2) one of the growth factors (Caov3, OVCA432, OVCAR4), or (3) none of the tested growth factors (OV90). Overall, Caov4 and OVCAR3 had the most similar response, with increased migration when treated with HB-EGF, NRG1β, or HGF; however, Caov4 had consistently greater wound closure. With respect to the individual growth factors, HGF had the broadest effect, with increased migration in Caov4, OVCA432, OVCAR3, and OVCAR4. Caov3, Caov4, and OVCAR3 all had increased wound closure when treated with HB-EGF, while only Caov4 and OVCAR3 were sensitive to NRG1β treatment. None of the cell lines studied exhibited increased wound closure after IGF1 treatment.

**Fig. 2:**
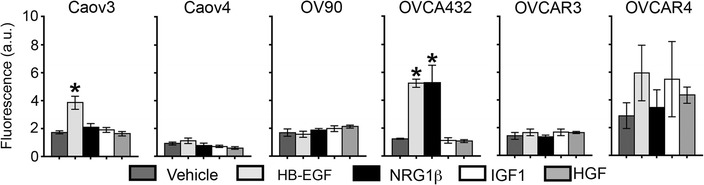
The effects of growth factors on single cell migration of HGSOC cell lines. HGSOC cell lines were seeded in transwells with 10 ng/mL HB-EGF, NRG1β, IGF1, or HGF in the bottom compartment. Following 48 h, cells that had migrated through the transwell were stained with Calcein-AM and the relative fluorescence was measured. For every cell line, an MDA-MB-231 positive control was performed in parallel; fluorescence for these cells was at least fivefold higher than for the HGSOC conditions. Data presented as the average ± SD, n = 3. *p < 0.05 compared to vehicle

Not surprisingly given the different biological mechanisms involved [30], differences in migration were observed between the wound and transwell assays. In contrast to the variability seen with wound closure, most of the cell lines exhibited minimal levels of baseline migration through transwells (Fig. 2). In addition, Caov4, OV90, OVCAR3, and OVCAR4 were unresponsive to all of the tested growth factors. Caov3 and OVCA432 had increased migration in response to HB-EGF, and OVCA432 was also sensitive to NRG1β. Consistent with the wound assay, IGF1 did not impact migration for any of the cell lines in the transwell assay. Interestingly, none of the cell lines had increased migration in response to HGF in transwell assays, despite HGF impacting wound closure in four of the cell lines (Fig. 1).

### MMP levels varied across HGSOC cell lines

**Fig. 3:**
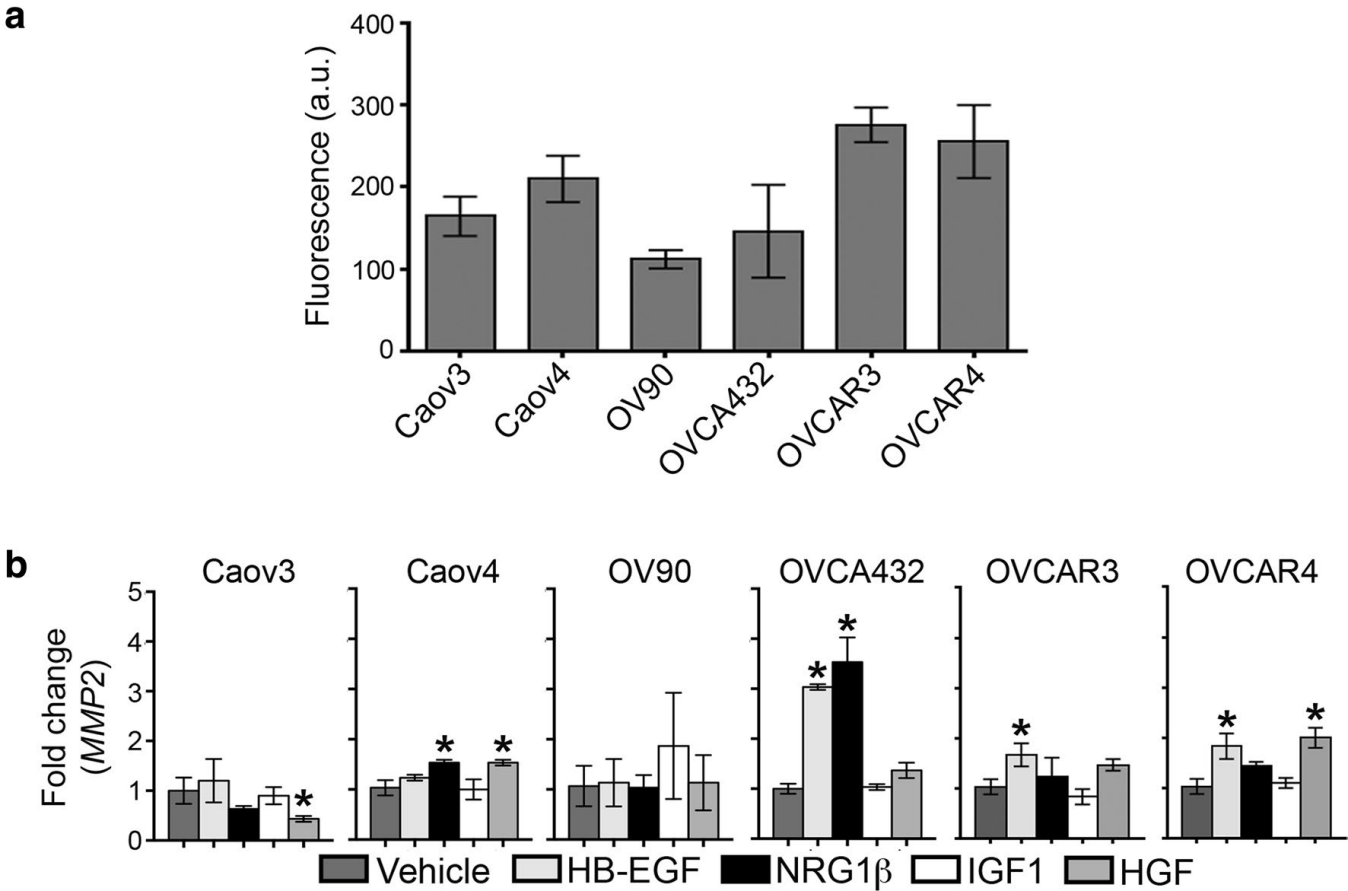
Growth factor-induced changes in *MMP2* expression of HGSOC cell lines. **a** Total baseline levels of active MMPs in conditioned media was determined to vary across the cell lines when assayed with a fluorogenic MMP substrate. **b** Treatment with 10 ng/mL HB-EGF, NRG1β, IGF1, or HGF for 24 h impacted *MMP2* expression in a subset of HGSOC cell lines. Expression levels were measured by qRT-PCR and fold change was determined using concurrently assayed *GAPDH* levels. Data presented as the average ± SD, n = 3. *p < 0.05 compared to vehicle

MMPs are a critical component of metastasis in solid tumors, enabling the degradation and remodeling of extracellular matrix as tumor cells invade through the basement membrane and stroma [31]. To examine variation in the remodeling characteristics of these HGSOC cells, conditioned media was first assayed to characterize the total level of MMPs secreted by the cell panel in the absence of growth factor stimulation (Fig. 3a). OV90 had the lowest levels of MMPs and OVCAR3 the highest levels. MMP2 has been shown to play a major role in mediating ovarian cancer cell attachment to the omentum and enabling tumor cells to invade through the extracellular matrix to establish further metastases [9, 10]. Therefore, we next examined the expression of *MMP2* and found that all of the cell lines had detectable levels (Fig. 3). Following growth factor treatment, cells had altered *MMP2* levels in response to (1) two growth factors (Caov4, OVCA432, OVCAR4), (2) one growth factor (Caov3, OVCAR3), or (3) none of the tested growth factors (OV90). In general, OVCAR3 and OVCAR4 showed the most similar patterns of sensitivity to the different growth factors. Most of these effects were modest, with only OVCA432 demonstrating increases in *MMP2* of greater than twofold after growth factor stimulation. With respect to the individual growth factors, our results demonstrated that HB-EGF increased *MMP2* levels in OVCA432, OVCAR3 and OVCAR4, and NRG1β increased *MMP2* expression in Caov4 and OVCA432. Similar to the results for migration (Figs. 1, 2), IGF1 did not significantly impact *MMP2* expression in any of the HGSOC cells tested. HGF increased *MMP2* in Caov4 and OVCAR4 but decreased levels in Caov3.

### Tumor cell proliferation in response to growth factors varied across HGSOC cell lines

**Fig. 4:**
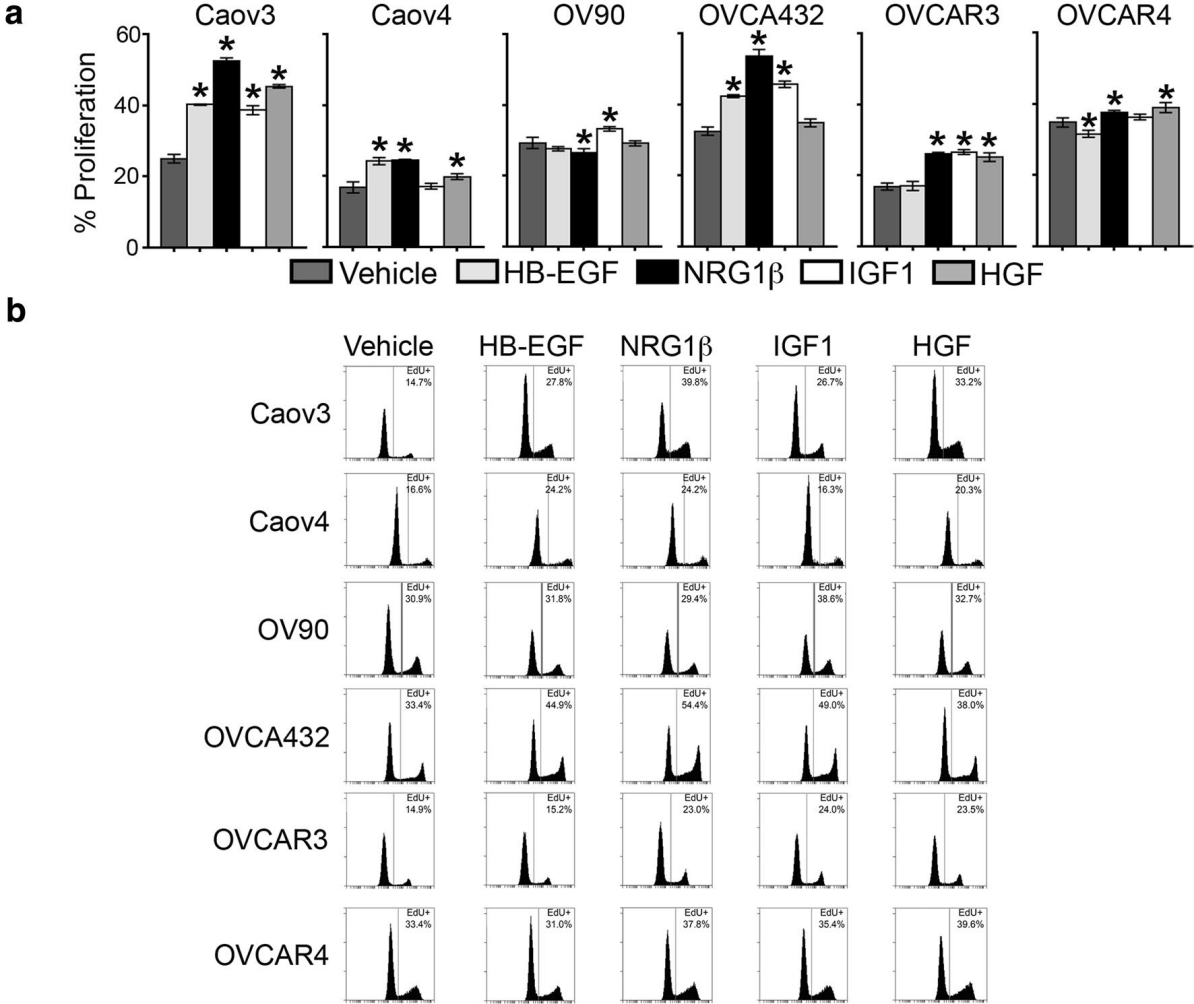
Baseline and growth factor-induced proliferation of HGSOC cell lines. **a** Baseline proliferation varied across the six HGSOC cell lines and treatment with 10 ng/mL HB-EGF, NRG1β, IGF1, or HGF for 24 h induced proliferation in a subset of these cell lines. Proliferation was determined by Click-iT EdU and flow cytometry. Data presented as the average ± SD, n = 3. *p < 0.05 compared to vehicle. **b** Representative flow cytometry histograms for each cell line-growth factor condition, with the percentage of EdU-positive cells (cells in S-phase) indicated

Elevated cell proliferation is associated with the advanced stages of HGSOC [13]. Therefore, we determined the percentage of cells in S-phase after treatment with vehicle, HB-EGF, NRG1β, IGF1, or HGF (Fig. 4). Despite the extended length of time in serum-free media, all cell lines continued to proliferate robustly and appeared healthy. A range of baseline levels of proliferation was detected (16.8 % for Caov4 to 34.8 % for OVCAR4). We observed that HGSOC cell lines had significantly increased proliferation following treatment with (1) all of the growth factors (Caov3) or (2) a subset of the growth factors (Caov4, OV90, OVCA432, OVCAR3, OVCAR4). In contrast to migration and *MMP2* expression, there were no cell lines insensitive to all four growth factors, although the effects on OV90 were modest. None of the members of the cell line panel demonstrated identical patterns of responsiveness; for example, despite comparable baseline proliferation rates, OV90 and OVCAR4 exhibited only small (<4 %) changes in proliferation regardless of which growth factor was used while OVCA432 was strongly sensitive to several growth factors. Of the growth factors, the effects of NRG1β were the most widespread, with large increases in proliferation for Caov3, Caov4, OVCA432, and OVCAR3, a slight increase for OVCAR4, and a slight decrease for OV90. HB-EGF significantly induced proliferation for Caov3, Caov4, and OVCA432, and a slight decrease in proliferation in OVCAR4 was seen. IGF1 induced proliferation in Caov3, OVCA432, and OVCAR3, with a slight increase for OV90. Finally, HGF increased proliferation for Caov3 and OVCAR3 with slight effects on Caov4 and OVCAR4.

### VEGF secretion in response to growth factors varied across HGSOC cell lines

**Fig. 5:**
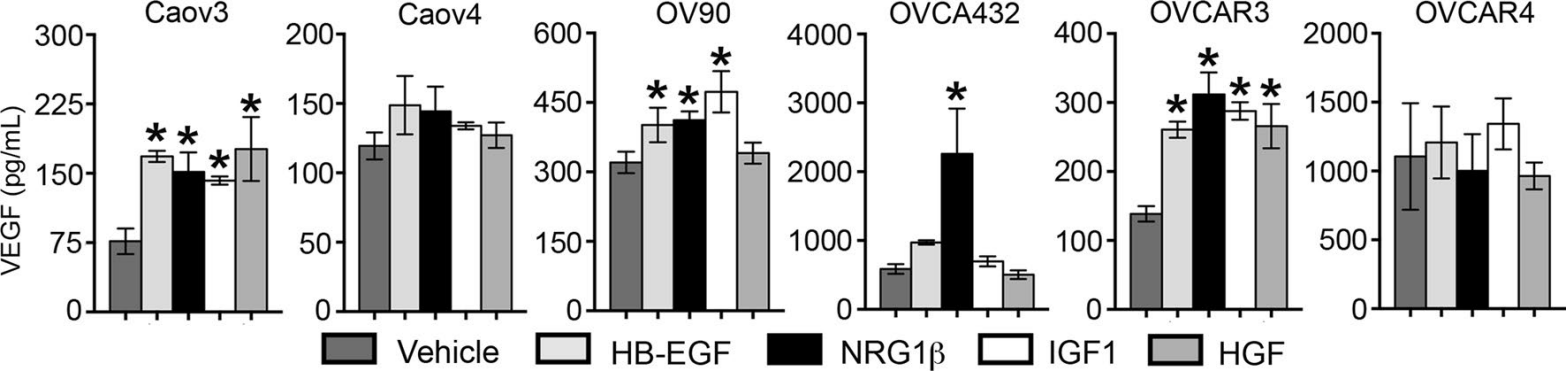
The impact of growth factors on VEGF secretion by HGSOC cell lines. HGSOC cell lines secreted varying baseline levels of VEGF and treatment with 10 ng/mL HB-EGF, NRG1β, IGF1, or HGF induced VEGF secretion in a subset of these cell lines. VEGF levels were determined by ELISA. Data presented as the average ± SD, n = 3. *p < 0.05 compared to vehicle. Note different scales on the y-axes

Production of VEGF by tumor cells and other cells in the microenvironment is essential for the development of new blood vessels to support tumor growth [32]; vessels that arise from VEGF-induced angiogenesis are often leaky, which in HGSOC results in the accumulation of large volumes of ascites fluid [14, 15]. Here, we analyzed the concentration of VEGF in conditioned media from six HGSOC cell lines after 24 h of stimulation with vehicle, HB-EGF, NRG1β, IGF1, or HGF (Fig. 5). In the absence of exogenous stimulation, all six cell lines secreted detectable levels of VEGF. These baseline concentrations varied greatly across the cell lines (ranging from 76.5 pg/mL for Caov3 to 1105.6 pg/mL for OVCAR4). Following growth factor treatment, VEGF levels increased in response to either: (1) all four growth factors (Caov3, OVCAR3), (2) a subset of growth factors (OV90, OVCA432), or (3) none of the tested growth factors (Caov4, OVCAR4). Overall, Caov3 and OVCAR3 showed the most similar response profile, with similar fold increases in response to each of the four tested growth factors. In contrast to the other behaviors studied, OV90 was responsive to growth factor stimulation, with increases in VEGF in response to HB-EGF, NRG1β, and IGF1. Among the growth factors, cells were most responsive to NRG1β, with increased levels of VEGF seen in Caov3, OV90, OVCA432, and OVCAR3. HB-EGF treatment induced VEGF secretion in Caov3, OV90 and OVCAR3; despite impacting unique receptor tyrosine kinase families, cells showed a similar sensitivity to IGF1 as they had to HB-EGF. HGF increased VEGF levels only in Caov3 and OVCAR3.

### Principal component analysis suggested HGSOC cell response to growth factors is dependent on the cell line

**Fig. 6:**
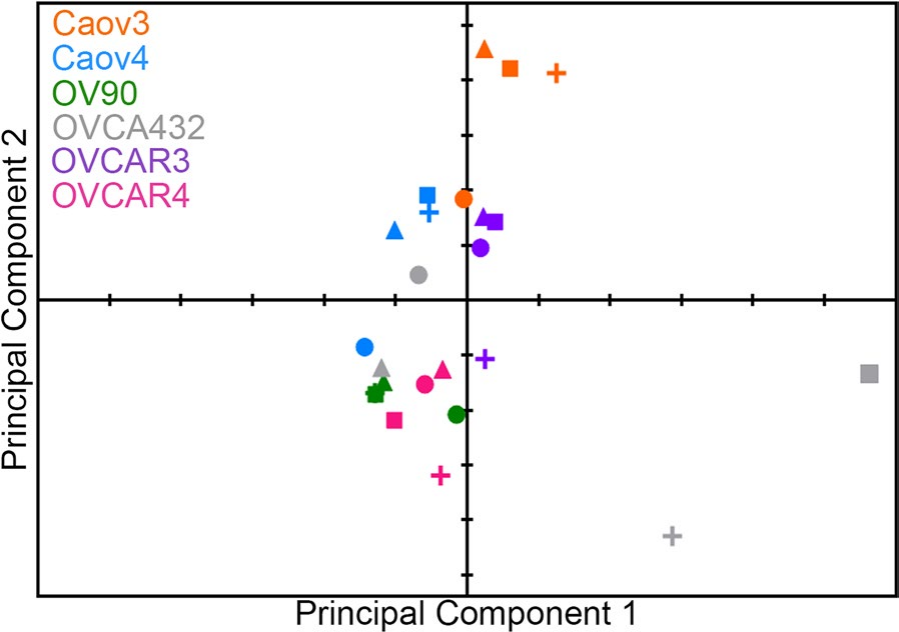
PCA of growth factor effects on HGSOC cell behaviors. Scores plot for the first two principal components of the analysis of growth factor-induced changes in HGSOC cell behavior. Each cell line-growth factor combination is represented by a* color *(for the HGSOC cell line) and* symbol* (for the growth factor: HB-EGF (*plus*), NRG1β (*square*), IGF1 (*circle*), and HGF (*triangle*))

To examine patterns in our data, principal component analysis (PCA) was performed on the growth factor-induced effects on migration, *MMP2* expression, proliferation, and VEGF secretion. As seen in the scores plot (Fig. 6), cell lines clustered together more closely than growth factor treatments. Cell lines were primarily separated by principal component 1; for example, Caov4, OV90, and OVCAR4 were generally unresponsive to the selected growth factors and projected negatively along this axis, while Caov3 and OVCAR3 were generally sensitive and projected along the positive axis. In contrast, all four growth factors were distributed across both principal component 1 and principal component 2.

We examined two potential reasons for this result. First, it is possible that the baseline behavior of the cells would influence the growth factor-induced response. For example, it is possible that cells with high basal production would not be able to produce additional VEGF. However, OVCA432 had the second highest basal level of VEGF (587 pg/mL), but was exquisitely sensitive to NRG1β, and nearly quadrupled the amount of VEGF produced (2258 pg/mL, Fig. 5). Additionally, baseline migration levels of Caov3 and Caov4 were similar, but Caov3 was substantially more responsive to HB-EGF (Fig. 1). Secondly, the level of the receptors for the selected growth factor could influence the activity of the network upon growth factor treatment. In some cases, baseline receptor levels (Additional file 1) could predict sensitivity to growth factor treatment. For example, OV90 had the second lowest level of ErbB3, the receptor for NRG1β, and was generally unresponsive to this growth factor (Figs. 1, 2, 3, 4). However, OV90 also had the highest levels of IGF1R and was insensitive to IGF1; indeed, in most cases there was no correlation between receptor level and growth factor response.

## Discussion

In order to determine if HGSOC cell lines with genomic profiles similar to the TCGA database demonstrate heterogeneity similar to that seen clinically, we examined differences in baseline migration, *MMP2* expression, proliferation and VEGF secretion, as well as responsiveness to HB-EGF, NRG1β, IGF1 and HGF. As most prior studies have utilized only one or two cell lines, comparisons of the baseline behaviors for these cell lines have not been previously reported. Additionally, only twelve of the 120 growth factor-induced endpoints in this study have been examined before [20, 25, 33–38]. Similar to the heterogeneity observed between HGSOC tumors, our analysis indicated that there was substantial variation between these cell lines, and PCA of the data set indicated that the cell behaviors were more strongly influenced by cell type than growth factor identity.

Our results indicated that each of four tested growth factors impacted several behaviors across the cell line panel. Of the 24 different cell line-endpoint combinations, HB-EGF and NRG1β significantly increased 14 endpoints and HGF increased 12 endpoints (each of these growth factors also resulted in one behavior that was slightly decreased). While the results were generally consistent with previous reports for the members of the cell panel [20, 25, 34, 36, 37] there were some noted differences relative to prior studies with SKOV3. For example, IGF1 did not stimulate migration in either of the assays, but has been shown to increase wound closure for SKOV3 [39]. Likewise, HGF had no effect on any cell line in the transwell assay but has been repeatedly shown to induce chemotactic migration in SKOV3 [38, 40, 41]. The diverse effects of the growth factors suggest that targeting one growth factor pathway will not be sufficient to abrogate tumor cell behavior and may help explain the inefficacy of receptor-targeted therapies in ovarian cancer [42–45]. One notable exception to this trend has been the promising results from clinical trials with anti-ErbB3 antibodies [46–48]. NRG1β stimulated proliferation and VEGF secretion in multiple cell lines suggesting this approach may successfully target multiple mechanisms in a larger population of patients.

Of the individual cellular behaviors examined, the selected growth factors had the strongest effect on proliferation, with three or more cell lines sensitive to each growth factor, and VEGF secretion, where each growth factor impacted at least two cell lines. This may of course result from the high sensitivity and precision of these assays and highlights the importance of considering assay choice when doing in vitro studies. The impact of assay choice was further exemplified by the striking differences observed between the wound and transwell assays for migration. While both assays are commonly used to measure cell migration, they examine different biological mechanisms as wound assays monitor collective cell migration and transwell assays assess single cell motility in response to chemotactic stimuli. Our results indicated that growth factors have a substantially stronger effect on collective migration versus single cell migration in the HGSOC cell lines. In addition, the effects of growth factor treatment on *MMP2* levels did not mirror the effects seen for migration, suggesting that unique therapeutic strategies may be required to control these different mechanisms of HGSOC metastasis.

It is of course important to compare our in vitro findings to data from clinical samples in order to determine if these cell lines that are representative of the HGSOC genotype [6] are also representative of its phenotype. The growth factors examined in this study have been previously suggested to have a role in HGSOC, are expressed in a high percentage of tumors, and are generally elevated compared to normal tissue [20, 49–53]. All growth factors were examined at a dose of 10 ng/mL, reflecting the concentrations reported for HB-EGF and HGF in ascites fluid of ovarian cancer patients [52, 54]. All cell lines proliferated when treated with vehicle; however, the extent of proliferation varied depending on the cell line (16.8–34.8 %). This spread was consistent with variation observed in proliferation in primary tumors (0.4–23.04 % [55]), although the absolute levels were higher. Additionally, all cell lines secreted detectable levels of VEGF in culture (76.5–1105.6 pg/mL), which was consistent with reports of serum levels in HGSOC (92–721 pg/mL [56]). MMPs are often expressed in HGSOC [57, 58]; thus, it was not surprising that all cell lines secreted detectable levels of MMPs. While it is not possible to directly relate these levels to reports from primary tumors, MMP levels varied between the examined lines and immunohistochemistry for MMP2 also showed variation between HGSOC samples [59].

Finally, even though a growth factor was capable of impacting a cell behavior in one cell line, there was no guarantee that it would impact another cell line, even if that other cell line was sensitive with respect to a different endpoint. For example, HB-EGF induced proliferation and migration in Caov3 but only proliferation for OVCA432. This is reflected in the clustering by cell type rather than growth factor in PCA. However, neither baseline cellular behaviors nor growth factor receptor levels were predictive of sensitivity. It is possible that variations in the levels of other components of the cellular signaling network besides growth factor receptors could be responsible for the observed heterogeneity. For example, it has been shown that when predicting cell response, dimerization patterns of ErbB1 with other ErbB receptors is important to consider [60], as is the ratio of IGF1 to IGF1R and the level of the IGF binding proteins [61]. Likewise, previous work from our lab demonstrated that it was necessary to incorporate the levels of both ligands and receptors when predicting ovarian cancer cell response to an ErbB-targeted inhibitor [62]. Ultimately, our analysis demonstrated that these six cell line models of HGSOC exhibit heterogeneity consistent with the disease. Our results also highlight the difficulty in developing targeted therapies for HGSOC since determining the target that will halt metastatic spread will not be as direct as simply identifying the growth factors or receptors present at the highest levels.

## Methods

### Cell lines and culture methods

Caov3 (HTB-75), Caov4 (HTB-76), OV90 (CRL-11732), OVCAR3 (HTB-161), and MDA-MB-231 were purchased from American Type Culture Collection (ATCC; Rockville, MD). OVCA432 [63] were obtained from Dr. R. Bast (MD Anderson Cancer Center; Houston, TX, USA). OVCAR4 [64] were obtained from the NCI Tumor Repository (Frederick, MD). HGSOC cell lines were authenticated by human short tandem repeat (STR) analysis at the Translational Research Initiatives in Pathology (TRIP) lab at the University of Wisconsin–Madison. Briefly, the analysis was conducted utilizing the Promega^®^ 16 HS reagent system, multiplex PCR amplification, capillary electrophoresis (ABI 3500) and GeneMapper (v.4.1) software. All HGSOC cell lines are reported to possess a mutation in *TP53* (Table 1). Cells were maintained at 37 °C in a humidified 5 % CO_2_ atmosphere. Caov3, Caov4, OV90, OVCA432, OVCAR4, and MDA-MB-231 were cultured in a 1:1 (v/v) ratio of MCDB 105 (Sigma-Aldrich; St. Louis, MO, USA) and Medium 199 (Corning; Corning, NY, USA) supplemented with 1 % penicillin/streptomycin (Life Technologies; Grand Island, NY, USA) and 10 % heat-inactivated fetal bovine serum (Life Technologies). OVCAR3 cells were cultured in RPMI 1640 (Corning) supplemented with 20 % heat-inactivated fetal bovine serum, 1 % penicillin/streptomycin, 1 % sodium pyruvate (Sigma), 0.3 % glucose (Corning), and 10 ng/mL insulin (Sigma).

**Table 1:** *TP53* mutation status of HGSOC cell lines utilized in this study

Cell line	*TP53* mutation
Caov3	406 C > T—Nonsense (Q136X) [6, 67, 68]
Caov4	440 T > A—Missense (V147D) [6, 67, 68]
OV90	643 A > C—Missense (S215R) [6, 67, 69]
OVCA432	830 G > T—Missense (C277F) [67, 70, 71]
OVCAR3	743 G > A—Missense (R248Q) [6, 70–73]
OVCAR4	388 C > G—Missense (L130 V) [6]

### Tumor cell migration

For wound assays, HGSOC cells were stained with 5 μM CellTracker™ Green CMFDA Dye (Life Technologies), plated in 48-well plates at cell densities empirically determined to result in 100 % confluency, and allowed to attach overnight. Specifically, Caov3, Caov4, and OV90 were plated at 132,000 cells/cm^2^, OVCA432 and OVCAR3 at 105,000 cells/cm^2^, and OVCAR4 at 121,000 cells/cm^2^. Cells were then rinsed with PBS and serum-free media was added. After serum starving for 24 h, a scratch was made in each well with a 200 μL pipette tip, cells were washed twice with serum-free media, and the wound was imaged with an Olympus IX51 Fluorescent Microscope (Olympus America Inc.; Center Valley, PA, USA). Cells were then treated with vehicle (0.1 % BSA in PBS) or 10 ng/mL recombinant human HB-EGF, NRG1β, IGF1, or HGF (Peprotech; Rocky Hill, NJ, USA) in serum-free media. Forty-eight hours post-treatment, the wound was imaged again, and the percent wound closure was measured using ImageJ (NIH; Bethesda, MD, USA).

Transwell assays were performed using polycarbonate Transwell^®^ inserts (#3422, Corning) with 8 μm pores. HGSOC cells were serum starved for 24 h, dissociated using TrypLE Select Enzyme (Life Technologies), and 150,000 cells/well were seeded in serum free media in the top compartments of the transwells inserted in 24-well plates. The bottom compartments contained 500 μL serum free media plus vehicle (0.1 % BSA in PBS) or 10 ng/mL HB-EGF, NRG1β, IGF1, or HGF. All experiments included a positive control of the highly invasive MDA-MB-231 breast cancer cell line [65] in the top compartment and 10 % serum-containing media in the bottom compartment. After 48 h of treatment, any cells which had migrated through the transwell into the bottom compartment were dissociated and stained for 30 min at 37 °C with 400 μL of a 4 μM Calcein AM (Life Technologies)/TrypLE dissociation solution. After dissociation and staining, 100 μL of each well was transferred to a black-walled, 96-well plate (Corning) and analyzed at 485 nm excitation and 520 nm emission using a Fluoroskan Ascent™ Microplate Fluorometer (Thermo Scientific; Waltham, MA, USA).

### MMP characterization

To characterize total levels of active MMPs, HGSOC cells were plated in 12-well plates at cell densities empirically determined to result in approximately 70 % confluency and allowed to attach overnight. Specifically, Caov3, Caov4, and OV90 were plated at 29,000 cells/cm^2^, OVCA432 and OVCAR3 at 24,000 cells/cm^2^, and OVCAR4 at 26,000 cells/cm^2^. Cells were then rinsed with PBS and serum-free media was added. After 48 h of serum starvation, conditioned media was collected and assayed at a 1:1 (v/v) ratio with 20 μM Mca-PLGL-Dpa-AR-NH2 fluorogenic MMP substrate (R&D Systems: Minneapolis, MN) in 96-well, black-walled plates. After a 24 h incubation at 37 °C, MMP activity was measured by quantifying fluorescent intensity at an excitation/emission of 320/405 nm. To determine the impact of growth factors on *MMP2* expression, cells were plated in 6-well plates at the same cell densities as the total MMP assay. After overnight attachment, cells were washed with PBS and serum-free media was added. Following 24 h of serum starvation, cells were washed with PBS and vehicle (0.1 % BSA in PBS) or 10 ng/mL HB-EGF, NRG1β, IGF1, or HGF was added in serum-free media. After 24 h of growth factor treatment, mRNA was isolated using the RNeasy Mini Kit (Qiagen; Hilden, Germany) according to manufacturer’s instructions and quantified on a NanoDrop 2000 Spectrophotometer (Thermo Scientific Pierce). cDNA was synthesized from 1 μg RNA using the Superscript III First-Strand Synthesis System kit (Life Technologies) according to manufacturer’s instructions. qRT-PCR was performed on 100 ng cDNA using SYBR Green PCR Master Mix (Life Technologies) and QuantiTect primers for *MMP2* (QT00088396, Qiagen) or *GAPDH* (QT00079247, Qiagen). The fold change in gene expression of *MMP2* was determined by the ΔΔC_t_ method relative to *GAPDH* expression.

### Tumor cell proliferation

HGSOC cells were plated in 12-well plates at the same cell densities as the *MMP2* expression assay and allowed to attach overnight. Cells were then rinsed with PBS and serum-free media was added. After serum starving for 24 h, cells were washed with PBS and stimulated with vehicle (0.1 % BSA in PBS) or 10 ng/mL HB-EGF, NRG1β, IGF1, or HGF in serum-free media. After 24 h of treatment, cell proliferation was quantified using the Click-iT^®^ EdU Alexa Fluor^®^ 488 flow cytometry assay (Life Technologies) according to manufacturer’s instructions. Cells were incubated with EdU for 6 h prior to sample collection and analyzed on a BD Accuri™ C6 flow cytometer (BD; Franklin Lakes, NJ, USA). Samples were gated for the EdU-positive population to determine the percentage of cells that entered S-phase during the EdU incubation.

### VEGF secretion

HGSOC cells were plated in 12-well plates at the same cell densities as the *MMP2* expression assay and allowed to attach overnight. Cells were then rinsed with PBS and serum-free media was added. After serum starving for 24 h, cells were washed with PBS and vehicle (0.1 % BSA in PBS) or 10 ng/mL HB-EGF, NRG1β, IGF1, or HGF was added in serum-free media. After 24 h of growth factor treatment, conditioned media was collected, and VEGF levels were determined by ELISA (#DY293B, R&D Systems) according to the manufacturer’s instructions.

### Principal component analysis

Principal component analysis (PCA, [66]) was performed on the resulting data matrix (composed of rows for each cell line-growth factor combination and columns of the levels of growth factor-induced response relative to vehicle-treated controls—i.e., percent increase in proliferation, percent increase in wound closure, fold change in fluorescence for transwell migration, fold change in *MMP2*, fold change in VEGF). The first principal component captured 47.0 % of the variation; inclusion of a second principal component increased this to 74.7 %. PCA was performed using SIMCA.P + v.12.0.1 (Umetrics; San Jose, CA, USA) with mean-centered and variance-scaled data.

### Statistical analysis

All data are presented as the mean ± standard deviation (n = 3). Additionally, all experiments were performed at least twice to ensure reproducibility. Statistical significance was determined using Dunnett’s with vehicle samples set as the control. All statistical calculations were performed with JMP 4.1 software (SAS Institute; Cary, NC, USA), with statistical significance set as p < 0.05.

## Additional file

10.1186/s12935-015-0263-4 Baseline receptor levels in HGSOC cell lines. Baseline levels of ErbB1, ErbB3, IGF1R, and MET receptors varied across the subset of six HGSOC cell lines. Shown are (A) representative blots and (B) quantification by densitometry relative to a concurrently-run control lysate. Data presented as the average ± SD, n = 3.

## Abbreviations

HGSOC: high-grade serous ovarian cancer
MMP2: matrix-metalloproteinase-2
VEGF: vascular endothelial growth factor
HB-EGF: heparin-binding EGF-like growth factor
NRG1β: neuregulin-1-beta
IGF1: insulin-like growth factor 1
HGF: hepatocyte growth factor
PCA: principal component analysis
