# Supplementary figures and images for: High-grade serous ovarian cancer cell lines exhibit heterogeneous responses to growth factor stimulation
Source: Cancer Cell Int. 2015 Dec 7;15:112. doi: 10.1186/s12935-015-0263-4 (PMC4672525; doi:10.1186/s12935-015-0263-4)

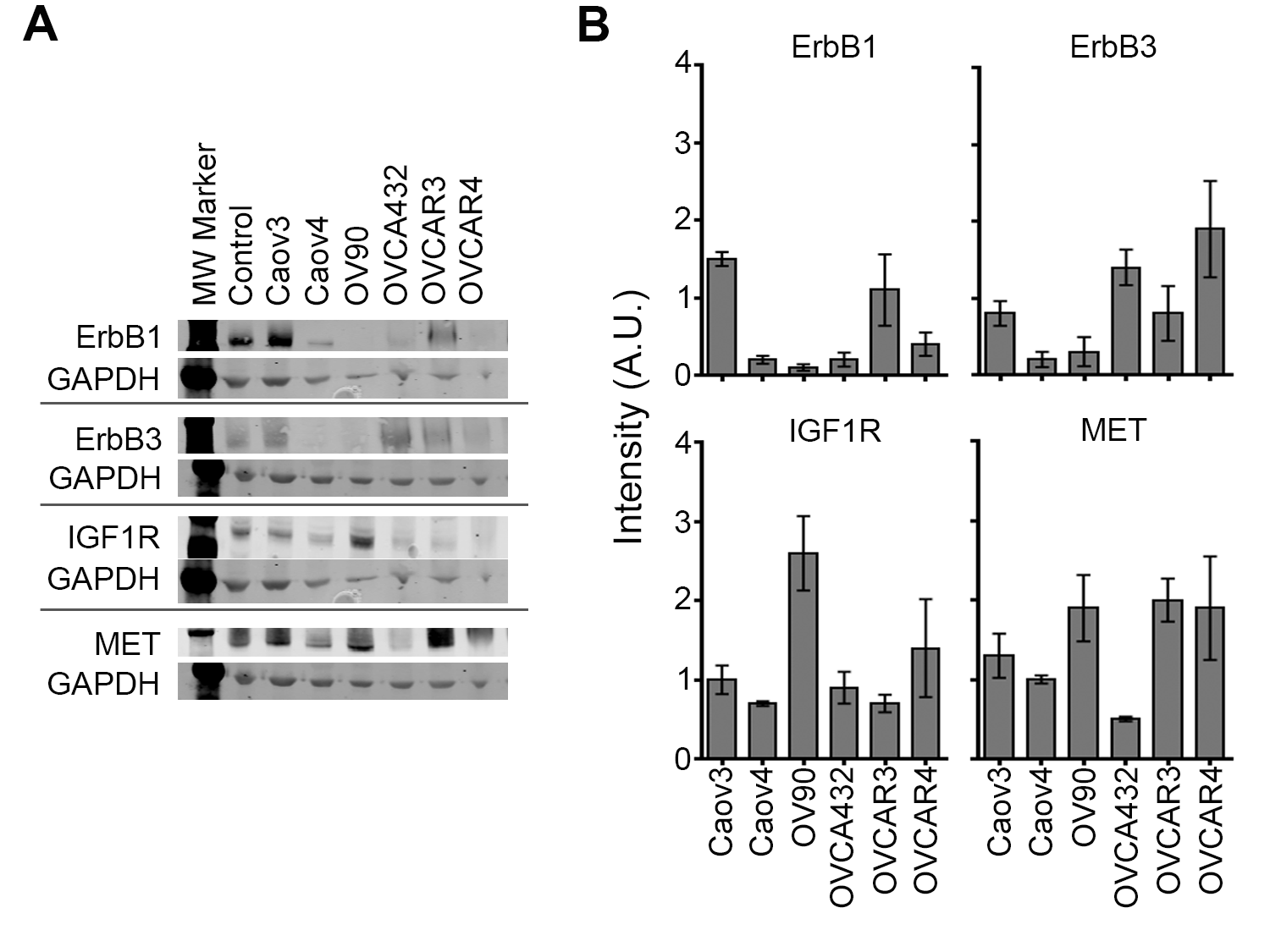

Supplement: Supplementary file 1 — 10.1186/s12935-015-0263-4 Baseline receptor levels in HGSOC cell lines. Baseline levels of ErbB1, ErbB3, IGF1R, and MET receptors varied across the subset of six HGSOC cell lines. Shown are (A) representative blots and (B) quantification by densitometry relative to a concurrently-run control lysate. Data presented as the average ± SD, n = 3. [file 12935_2015_263_MOESM1_ESM.tif]
